# Characterizing the Impedance Properties of Dry E-Textile Electrodes Based on Contact Force and Perspiration

**DOI:** 10.3390/bios13070728

**Published:** 2023-07-13

**Authors:** Vignesh Ravichandran, Izabela Ciesielska-Wrobel, Md Abdullah al Rumon, Dhaval Solanki, Kunal Mankodiya

**Affiliations:** 1 Department of Electrical, Computer and Biomedical Engineering, University of Rhode Island, Kingston, RI 02881, USA; vignesh_ravi@uri.edu (V.R.); mrumon@uri.edu (M.A.a.R.); dhaval_solanki@uri.edu (D.S.); 2 Department of Textiles, Fashion Merchandising and Design, University of Rhode Island, Kingston, RI 02881, USA; iciewrobel@uri.edu

**Keywords:** biopotential electrodes, e-textile electrodes, technical embroidery, electrode impedance characterization, smart textiles, biosignals

## Abstract

Biopotential electrodes play an integral role within smart wearables and clothing in capturing vital signals like electrocardiogram (ECG), electromyogram (EMG), and electroencephalogram (EEG). This study focuses on dry e-textile electrodes (E1–E6) and a laser-cut knit electrode (E7), to assess their impedance characteristics under varying contact forces and moisture conditions. Synthetic perspiration was applied using a moisture management tester and impedance was measured before and after exposure, followed by a 24 h controlled drying period. Concurrently, the signal-to-noise ratio (SNR) of the dry electrode was evaluated during ECG data collection on a healthy participant. Our findings revealed that, prior to moisture exposure, the impedance of electrodes E7, E5, and E2 was below 200 ohm, dropping to below 120 ohm post-exposure. Embroidered electrodes E6 and E4 exhibited an over 25% decrease in mean impedance after moisture exposure, indicating the impact of stitch design and moisture on impedance. Following the controlled drying, certain electrodes (E1, E2, E3, and E4) experienced an over 30% increase in mean impedance. Overall, knit electrode E7, and embroidered electrodes E2 and E6, demonstrated superior performance in terms of impedance, moisture retention, and ECG signal quality, revealing promising avenues for future biopotential electrode designs.

## 1. Introduction

The longitudinal monitoring of biopotential signals like electrocardiogram (ECG), electromyogram (EMG), and electroencephalogram (EEG) enables a wide range of use for cases such as the remote assessment of medical disorders [1], athletic performance [2], and brain–computer interfaces [3]. Contact-based biopotential-sensing electrodes such as Ag/Ag-Cl electrodes typically use gel or adhesive which provides minimal skin–electrode impedance [4]. While such electrodes are well-suited for short recording sessions, the gel and adhesives used can provoke skin irritation and often require skin abrasion and hair removal [4]. During long-term use, the gels within such electrodes dehydrate and adhesives weaken resulting in a decrease in signal quality [5].

Dry e-textile electrodes, in contrast to gel-based electrodes, do not use conductive gels and can be fabricated using fabric materials and fibers, enabling easy integration into daily used wearable textiles such as clothing [6]. They offer a significant improvement in comfort, making them a suitable choice for long-term monitoring. However, the performance of such dry electrodes is strongly influenced by extraneous factors such as skin coupling forces and perspiration encountered in daily life conditions. The effectiveness of a dry e-textile electrode structure and material choices can be evaluated using skin–electrode impedance measurements at different frequencies. The evaluation of such electrodes is typically conducted on human skin and is susceptible to inter-participant and intra-participant variability since skin impedance varies over time [7,8]. Alternatively, agar and gelatine skin phantoms can be used to measure the electrical characteristics of different electrodes [9,10]. Though exposure to perspiration initially reduces impedance, effects such as surface oxidation and residue buildup eventually increase it [11]. In this work, we design different e-textile dry electrodes and characterize their impedance using agar skin phantom under varying contact forces before and after exposure to synthetic perspiration.

**Figure 1:**
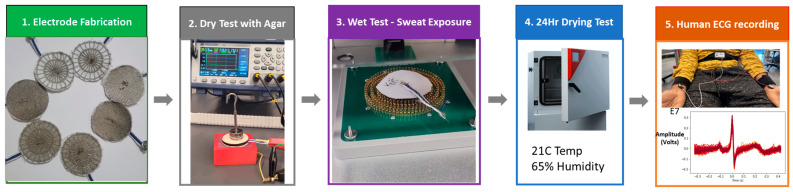
A graphical overview of our textile electrode evaluation study showcasing fabrication, impedance tests under different conditions, and human ECG acquisition.

Our specific contributions, visualized in Figure 1, are detailed as follows:

- *Dry e-textile electrode design and fabrication*: E-textile electrodes (six embroidered [E1–E6], one knit [E7]) were fabricated with different structural characteristics (uniform layout vs non-uniform layout). The changes in electrode layout and stitch density can influence impedance due to factors such as contact area, number, and length of conduction pathways available to the electrode.
- *Electrode impedance characterization*: To understand the influence of contact force and electrode impedance, electrode characterization uses a conductive agar-based skin phantom. We describe the process of creating the agar skin phantom using a 3D-printed mold with an integrated electrode.
- *Perspiration exposure analysis*: Prior inquiries have characterized the influence of contact force and electrode shape on impedance. The effect of controlled perspiration exposure, however, has seen limited exploration. We report of changes in impedance for different dry e-textile electrodes under varying loads (weights) before and after exposure to synthetic perspiration using a moisture management tester.
- *ECG signal acquisition*: The evaluation of the signal-to-noise ratio for ECG signals acquired using these different dry electrodes and gel electrodes.

## 2. Background and Related Works

Electrochemical cell activity results in biopotentials caused by changes in ion concentrations within the body. Commonly known biopotential signals generated by the heart, muscles, and brain are ECG, EMG, and EEG, respectively. Such cells, upon stimulation, cause an action potential, the gross summation of which results in the biopotential signals. The signals are characterized by their low amplitude (microvolts to millivolts), frequency (100 Hz to 1 kHz), and the presence of noise and artifacts. During biopotential signal acquisition, recorded signals might be overpowered by noise and other artifacts, which can be classified into three types: (1) electromagnetic (e.g., power line interference and radiofrequency sources), (2) motion (e.g., poor electrode contact and mechanical vibrations), and (3) physiological artifacts (e.g., respiration in ECG and eye blinks in EEG). The low-amplitude biopotential signals also have high impedance during measurement, requiring specialized amplifiers called biopotential amplifiers.

### 2.1. Skin–Electrode Impedance

**Figure 2:**
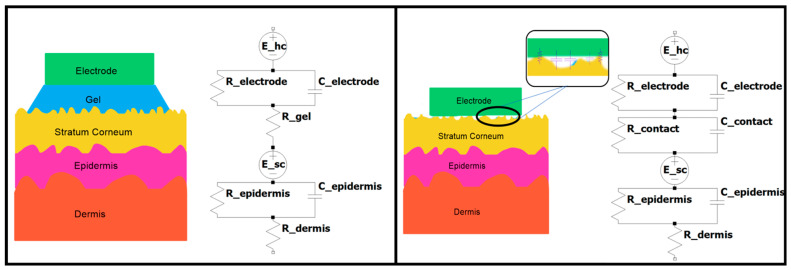
Skin–electrode equivalent circuit models adapted from [14]. Gel electrode (**left**) and dry electrode (**right**).

Biopotential signals are detected by electrodes that act as an interface between the body and measurement circuits. A low impedance between the skin and electrode is ideal since it improves the signal-to-noise ratio (SNR) and reduces the influences of external interference or artifacts [12]. The skin–electrode interface includes different layers of the skin and electrode that can be electrically modeled as equivalent circuits shown in Figure 2 for dry and gel electrodes. Dry electrodes have slightly different skin impedance models than gel electrodes caused by cumulative air gaps and hairs resulting in both capacitive and resistive coupling with skin. On the other hand, gel-based and semi-dry electrodes enable resistive coupling [13]. This base model can be expanded to include perspiration glands and other ducts that can be represented as a parallel resistor–capacitor (RC) network with sources within the epidermis section [14].

### 2.2. Comparison of Dry Electrodes

Despite the use of gel electrodes in clinical practice, they introduce several issues when used for long-term monitoring. The gel and adhesives used in such electrodes can cause skin irritation, and the drying of the gel reduces the signal quality over time [5]. For improved user comfort, dry electrodes are preferred for long-term monitoring [15]. While this work focuses explicitly on textile electrodes, a summary of different dry electrode types alongside their characteristics can be seen in Table 1.

Frequency-based skin–electrode impedance analysis shows that both silver-polymer- and PEDOT:PSS-based electrodes have electrode–skin impedance in the range of mega ohm at lower frequencies [16]. Another study shows that the impedance of the graphene-coated electrodes have electrode–skin impedance in the range of kilo ohm at lower frequencies [17] which is lower than the silver-polymer- and PEDOT:PSS-based electrodes. Recently, graphene-based flexible sensors and electrodes have been created using bamboo fibers using nanocomposite paper [22]. Despite its advantages such as lower impedance and high mechanical stability, such graphene electrodes have limited adoption outside of research settings at the present due to its high cost and complex fabrication process [23]. In contrast, silver polymer yarns are widely used to create such biopotential electrodes both in research and commercial settings [9]. Silver provides high conductivity, biocompatibility, and stability in comparison to other metals [20,24]. Larger-sized dry electrodes made from silver polymer yarn showcase surface impedance characteristics similar to graphene electrodes [25]. It is important to note that the structural characteristics and layout of such electrodes influences electrode impedance and its stability through changes in conduction paths [26,27]. Dry e-textile electrodes can be created through a multitude of approaches, such as weaving, embroidery, and knitting, with differing properties and characteristics. Woven electrodes are created by interlacing two sets of conductive yarns (warp and weft) into a flexible structure using looms [18]. Combining the yarns causes considerably higher tension in warp yarn compared to weft yarn, thus requiring a snug fit to reduce noise associated with loose threads [18]. Knitted electrodes are created as a continuous loop made using conductive yarn interlocked with previous rows resulting in a structure capable of conducting both within and across rows [20]. This approach produces highly stretchable and breathable electrodes that can be used to acquire cardiac (ECG) and ocular (EOG) biopotential signals [28]. Knitted electrode impedance can be reduced through increased size, contact pressure, conductive yarn density, and surface coarseness [20].

Embroidered electrodes are created by integrating conductive thread over a suitable base fabric or readymade garment using a needle [19]. This approach provides a high degree of flexibility in the layout of electrodes while enabling easy integration with electronic components and circuits [29]. Like knitted electrodes, the impedance of embroidered electrodes is reduced through an increase in size, density, and length [30].

Perspiration or human sweat is another relevant physiological factor that affects the impedance of such contact-based textile electrodes, especially in ambulatory monitoring conditions. Perspiration is moderately acidic to mildly alkaline, with a pH typically between 5.5 and 8.0 [31]. It causes a reduction of electrode impedance in the short term by enabling better contact between the electrode and stratum corneum [14]. However, over time, it can cause oxidation within the metalized yarns and threads [11]. Prior work has evaluated the impedance characteristics of dry e-textile electrodes on the basis of their structure and contact force without considering the influence of perspiration exposure [20,30]. Inquiries evaluating the effect of moisture exposure rely on either repeated washing or immersion in a saline solution for a set time period, though the effect of controlled drying was not studied [11,32]. Thus, there is a need to inquire about the influence of controlled perspiration exposure on the impedance of dry e-textile structures, both immediately after exposure and after controlled drying.

## 3. Materials and Methods

In this section, we describe the fabrication approach we employed to create embroidered knit electrodes and the agar skin phantom. We also explain the experimental methods of testing the influence of contact forces and perspiration exposure based on agar skin phantom impedance.

### 3.1. Dry E-Textile Electrode Fabrication

**Figure 3:**
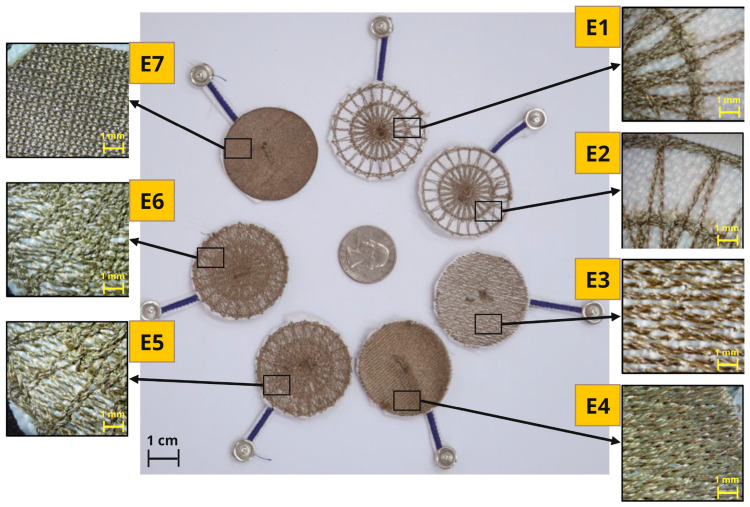
Seven different circular dry e-textile electrodes were evaluated in this study, along with zoomed images showing their structures. (Yellow embroidered [E1–E6], green knitted [E7].)

Seven different dry e-textile electrodes (6 embroidered [E1–E6], 1 knit [E7]) that were designed and evaluated in this study had a circular shape with a diameter of 40 mm. Their thread/yarn structure can be seen in Figure 3. All dry electrodes were created on a densely woven, non-conductive fabric made of polyester that does not retain water, providing a stable base for embroidery and perspiration exposure testing. The embroidered electrodes were fabricated using the F-head of an industrial technical embroidery machine (ZSK JGVA 0109, ZSK Stickmaschinen GmbH, Krefeld, Germany). Polyamide core and silver-plated Madeira HC40 (<100 Ω/m) threads were used to create different electrode patterns based on design templates in the EPCwin software. The embroidered electrode designs have different stitch patterns that result in varying structural and electrical characteristics.

Cross ball electrodes were embroidered sequentially over a foundation layer of straight stitches (spokes) radiating from a center point expanding outward with an offset between previous layers resulting in concentric ring structures with a hub in the middle. We created cross ball electrodes with a stitch length of 1 mm (E1) and a stitch length of 2 mm (E2), resulting in a higher stitch density in E1 and a lower stitch density in E2. The comfort fill electrode (E3) covers a circular area with a single layer of silver polymer thread along the horizontal axis using straight stitches. The comfort fill plus electrode (E4) covers a circular area with two layers of silver polymer thread along both the horizontal and vertical axis using straight stitches. These two embroidered electrode designs have an even layout with similar stitch density within the electrode, E4; due to the additional layer, it had a higher stitch density than E3. By stitching the cross ball patterns (E1, E2) over a base layer of comfort fill pattern (E3), hybrid electrodes were created for 2 mm (E5) and 1 mm (E6) stitch lengths.

**Figure 4:**
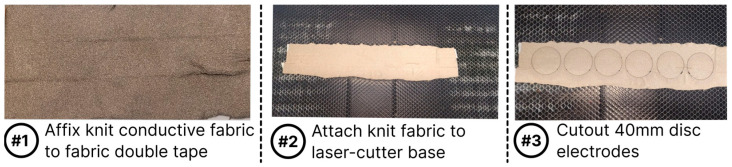
Laser cutting process used to create circular knit electrodes (E7).

We cut out circular knit electrodes (E7) with a diameter of 40 mm from Medtex 180 silver-plated nylon fabric with high surface conductivity (<5 Ω/sq cm). The glowforge computerized laser cutter was used to cut the electrodes from the fabric. The knit conductive stretch fabric was taped to the edges of the laser cutter base, as seen in Figure 4. Since this study seeks to understand the effect of contact forces on the electrode impedance, it is vital to ensure any force applied is distributed evenly. The ideal type of connector for this study should ensure reliable electrical contact while also maintaining even distribution of force applied by load weights. We chose textile lead connectors over the regular snap lead connectors for this study. We embroidered the textile lead connector separately using the industrial embroidery machine with running stitches of conductive thread that linked two 4 mm conductive pads. The conductive thread channels were insulated using navy-blue polyester thread that formed satin stitches with 2 mm width over the conductive running stitches. In order to obtain measurements from the textile electrodes, the textile lead connectors were stitched to the electrodes. We used the Madeira HC40 conductive thread to stitch the conductive pads of the lead connector in the middle of each dry electrode using a desktop sewing machine (Brother SE1900). Table 2 details the thickness associated with the dry electrode measured using a fabric thickness gauge across 15 different points in the electrode.

**Figure 5:**
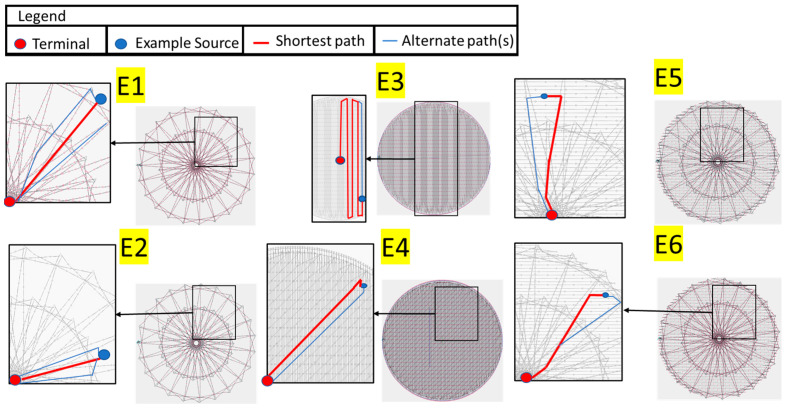
Example of conduction paths available within the embroidered electrodes (E1–E6).

All seven electrodes were identified based on the relative density of conductive threads and surface structures. The dry e-textile electrodes evaluated in this study were composed of different structures and consequently had differing skin contact areas and conduction paths, as seen in Figure 5. Electrodes E1 and E2 were uniquely characterized by their concentric structures or “cross balls” comprised of a greater density towards the central region. Contrarily, electrodes E3 and E4 were constructed with numerous stitches, formed in a back-and-forth pattern. Particularly, E3 is filled exclusively with sewing threads running in a single direction, whereas E4 is composed of bidirectional running threads, thus yielding a higher conductive thread density. The design principles of E1 through E3 were combined to create electrodes E5 and E6. These electrodes incorporated an overlapping design of cross ball and comfort fill structures, consequently increasing stitch depth and producing more cross-connections relative to electrodes E1 and E2. Upon comparison of contact area, electrodes E4 (comfort fill plus) and E7 (knit) were observed to have the most substantial regions filled with conductive yarn and compactness among the specimens, followed by E5 and E6. Such electrodes were expected to show minimal permeability of water and, in fact, were expected to hold on to the perspiration that was absorbed during exposure.

### 3.2. Agar-Based Skin Electrical Phantom

**Figure 6:**
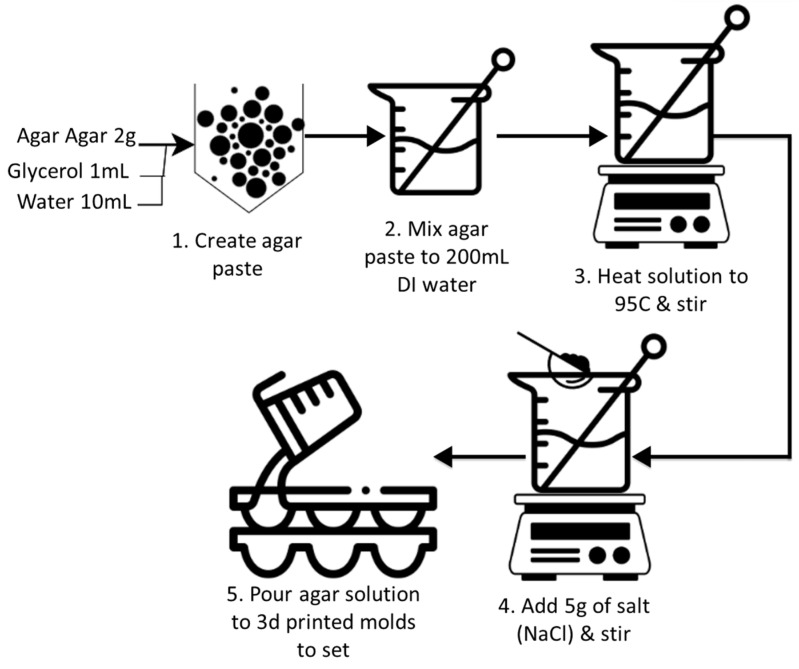
The process to create a conductive agar phantom.

The variability of skin–electrode impedance between and within individuals complicates the evaluation of different electrode types [7]. Further, the density of human eccrine glands (perspiration) varies from 70 to 140/cm^2^ throughout the torso and limbs, which can affect the impedance of different electrode types during simultaneous comparison studies. Therefore, a standardized test setup is required to evaluate the contact impedance characteristics of different electrode types. For this, skin phantoms can be created using agar or gelatine to evaluate biopotential sensing or stimulation electrodes with electrical properties mimicking skin [9,10,20,33]. They provide stable electrical characteristics unlike human skin enabling standard characterization of such electrodes. Owada et al. indicated that the electrical properties of such phantoms made with 3D-printed mold with integrated Ag/AgCl electrodes can be fine-tuned by adjusting the concentration of NaCl added to the mixture [10]. In this work, we utilized a similar process, as seen in Figure 6, to create an agar skin phantom with an integrated Ag/AgCl electrode to evaluate our dry e-textile electrodes.

**Figure 7:**
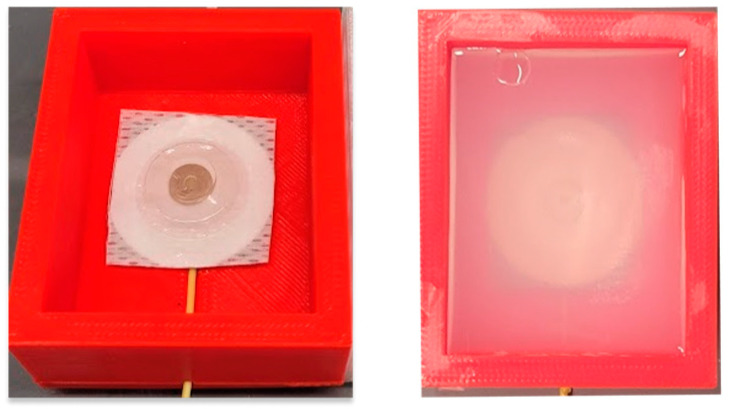
3D-printed mold with integrated electrode before adding agar (**left**). Conductive agar phantom after setting (**right**).

The process involves first mixing 2 g agar (lab-grade) with 1 mL of glycerol and 10 mL of deionized (DI) water to create a paste. Next, the paste is dissolved in 200 mL DI water which is heated to 95 °C while being stirred. Once the temperature reaches 95 °C, 5 g of sodium chloride (Sigma Aldrich, St. Louis, MO, USA) is stirred into the solution as it cools to room temperature. The solution is then shortly added to the 3D-printed electrode molds and allowed to solidify in isolation for 48 h at room temperature. The 3D-printed mold used was a cuboid (outer dimensions: 60 × 50 × 25 mm) with a hollow interior (inner dimensions: 50 × 40 × 20 mm) with an inner cavity for the internal gel electrode snap and cable, as seen in Figure 7. The agar solution is poured onto the mold and left to cool and solidify for 48 h before testing.

### 3.3. Dry Impedance Test

We conducted the dry impedance test to understand the influence of contact force on different biopotential electrodes (dry and gel) using a conductive agar phantom. All impedance measurements were conducted using a Bench LCR meter (BK 891) for the frequency range of 20 Hz to 5 KHz in increments of 10 Hz and logged in a .TXT file. Each electrode type was placed in the middle of the agar skin phantom one after another under different contact force conditions while the impedance measurements were acquired. To ensure the electrodes made even contact over the surface of the phantom, a 1/8” maple plywood disc with a diameter of 40 mm and weighing 3.6 g was placed over the electrode to distribute force evenly. Standard slotted weights were placed over the plywood disc in 10 g increments from 10 g to 40 g. The forces associated with each weight applied on each electrode can be seen in Table 3.

**Figure 8:**
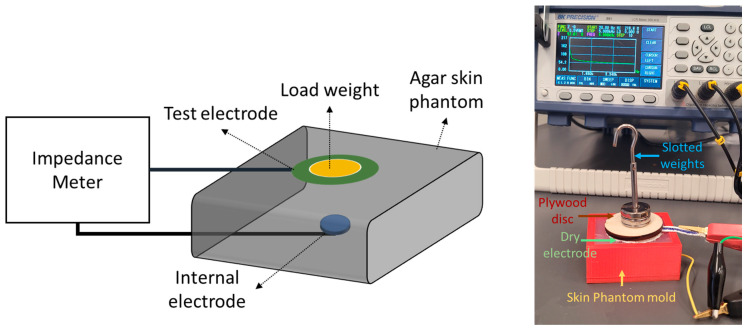
Dry electrode impedance evaluation setup (**left**). Dry electrode skin impedance measurement phantom under 43.6 g load (**right**).

Figure 8 shows the impedance measurement setup with a dry electrode under load over a conductive agar phantom. Between each test session, the conductive agar phantom was immersed in deionized water.

### 3.4. Controlled Perspiration Exposure Test

Each type of dry electrode was exposed to the same amount of perspiration using the SDL Atlas Moisture Management Tester (MMT model M290) for a duration of 60 s before performing the impedance test using the skin phantom [34,35]. The synthetic alkaline perspiration solution was prepared based on the established ISO 105-E04 standard protocol [36]. It is worth noting that, while this standard protocol is primarily employed to evaluate colorfastness (i.e., characterizing a material’s color resistance to fading or running) in conventional textile materials, previous research has successfully applied this method to investigate the effects of artificial perspiration on conductive textile materials such as yarn and fabric. Throughout the experimentation, we maintained a controlled testing environment at a temperature of (37 ± 2) °C. The artificial alkaline precipitation sample (pH 8 ± 0.2) consists of the following chemicals sourced from Sigma Aldrich.

1. L-histidine monohydrochloride monohydrate (0.5 g) [C6H9O2N3·HCl·H2O]
2. Sodium chloride (5 g) [NaCl]
3. Disodium hydrogen orthophosphatedodecahydrate (5 g) [Na2HPO4·12H2O]
4. Disodium hydrogen orthophosphate dihydrate (2.5 g) [Na2HPO4·2H2O]

**Figure 9:**
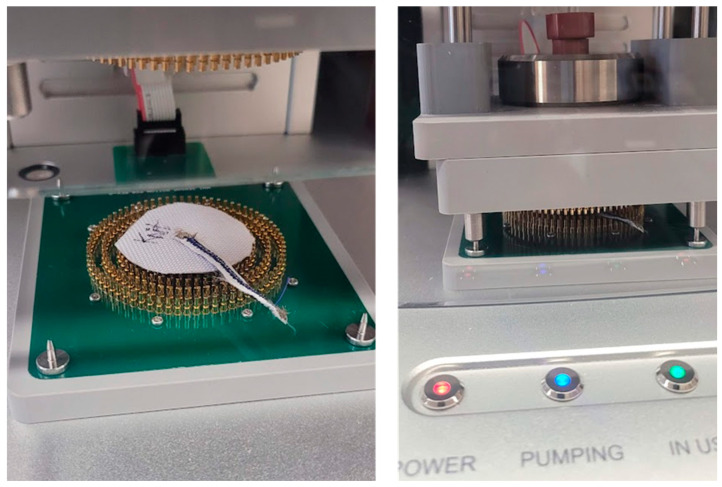
Perspiration exposure test setup (**left**). Knit electrode before perspiration exposure (**right**). Knit electrode during perspiration exposure.

Next, the impedance sweep measurements were performed for different weights, as presented in Table 2. Figure 9 shows a dry electrode exposed to synthetic perspiration in the MMT system. The perspiration was doused gradually through a dosage pump in the MMT for two minutes. After acquiring the impedance measurements, the dry electrodes were kept inside the Datacolor conditioning cabinet at 21 °C and 65% relative humidity for 24 h [37]. Another batch of impedance sweep measurements was conducted for different weights after the same period on the skin phantom.

### 3.5. Electrocardiogram (ECG) Signal-to-Noise Test

**Figure 10:**
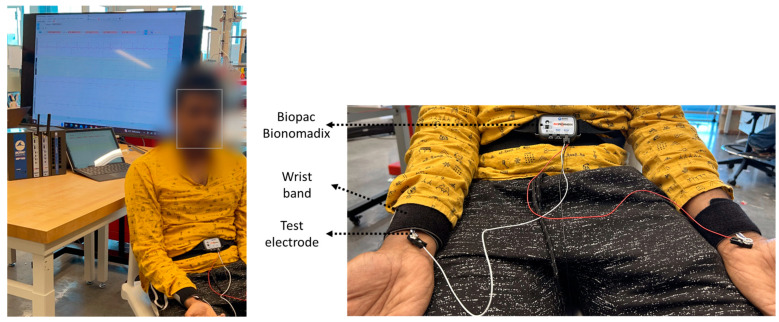
Single-lead Biopac ECG acquisition setup for testing different electrodes (**left**). Complete recording setup including Biopac MP160 and Windows computer (**right**). Close-up image of ECG acquisition setup.

In order to understand how the electrodes perform on biopotential signals, we acquire a single-lead ECG signal using the Biopac Bionomadix acquisition system at 2 KHz [38] sampling rate. A two-minute duration ECG signal for each electrode type was acquired from both wrists of a single 27-year-old male participant, as seen in Figure 10. The participant was instructed to stay seated and limit their motion during recording. The electrodes were supported by a wristband. Recording files were analyzed in Python using the Neurokit2 package [39]. The ECG signals were segmented to identify P, Q, R, S, and T sections for SNR calculations. The signal-to-noise ratios of different electrode types were calculated using two methods, (i) based on the power between the QRS section compared to the TP section (Equation (1)) and (ii) based on the mean and standard deviation of the ECG signal (Equation (2)).

(1) $${SNR}_{QRS/TP}=10\log_{10}\left(\frac{{Power}_{QRS}}{{Power}_{TP}}\right)$$

(2) $${SNR}_{Reg}=10\log_{10}\left(abs(\frac{{Mean}_{ECG}}{{Std}_{ECG}})\right)$$

## 4. Results

In this section, we described our findings and results from testing on the influence of contact forces and perspiration exposure on different dry electrode impedance.

### 4.1. Dry Impedance Test

**Figure 11:**
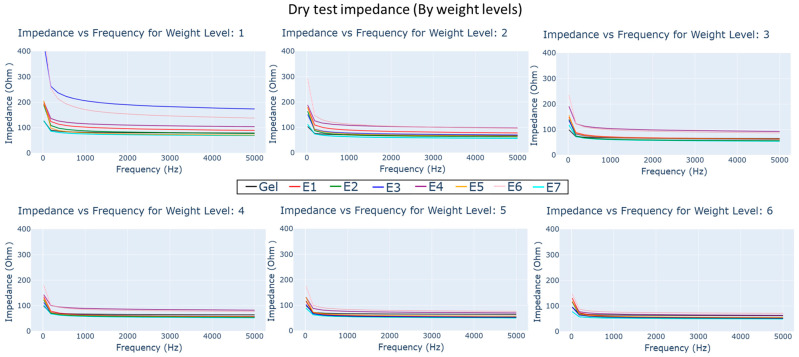
Impedance vs. frequency plots for dry and gel electrode types under different load weight conditions.

The impedance–frequency plots of electrodes under different load weights can be seen as subplots in Figure 11. The colors in the plot refer to different electrode types; it can be observed that in general the impedance reduces with higher frequency and weight levels (expanded further in Section 5).

### 4.2. Wet Impedance Test

**Figure 12:**
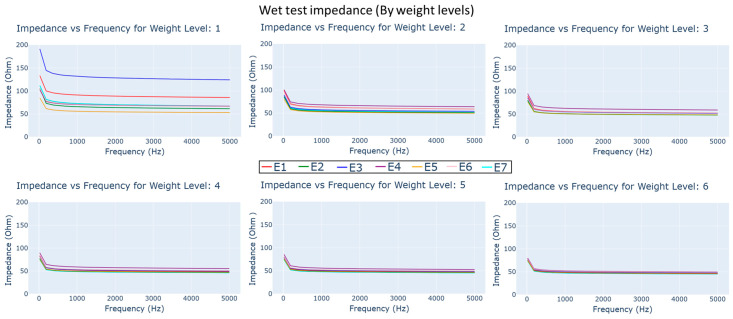
Impedance vs. frequency plots for different dry electrode types under different load weight conditions immediately after exposure to synthetic perspiration.

The impedance–frequency plots of electrodes exposed to synthetic perspiration for 60 s in MMT under different load weights can be seen in Figure 12. While the impedance still appears to reduce with higher frequency and weight level, there appears to be a sharp reduction compared the dry impedance test.

**Figure 13:**
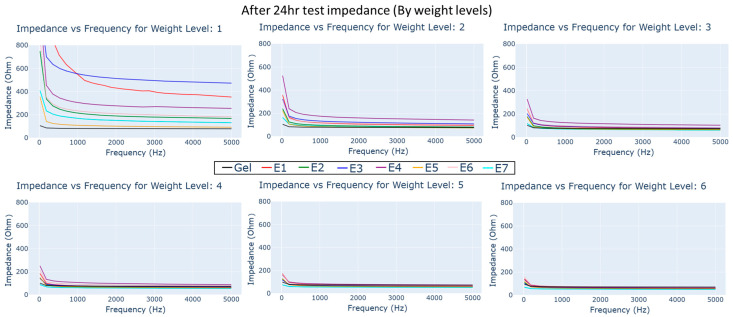
Impedance vs. frequency plots for dry and gel electrode types under different load weight conditions 24 h after exposure to synthetic perspiration.

The electrodes were allowed to dry for 24 h in the conditioning chamber. The impedance–frequency measurements were conducted under different load weights, as seen in Figure 13. The reduction in impedance with weight and frequency can still be observed; however, there is an increase in overall impedance across all frequency and weights compared to the dry and wet impedance tests.

**Figure 14:**
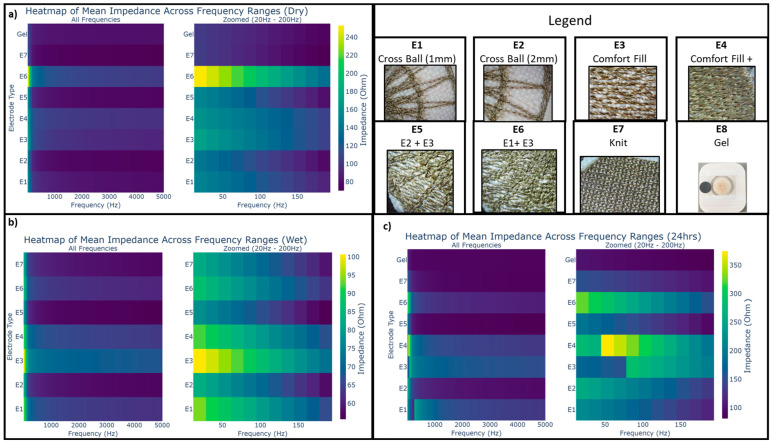
Mean impedance vs. frequency heatmap for dry and gel electrode types under different test conditions (**a**) dry impedance, (**b**) immediately after perspiration exposure, and (**c**) after perspiration exposure.

**Figure 15:**
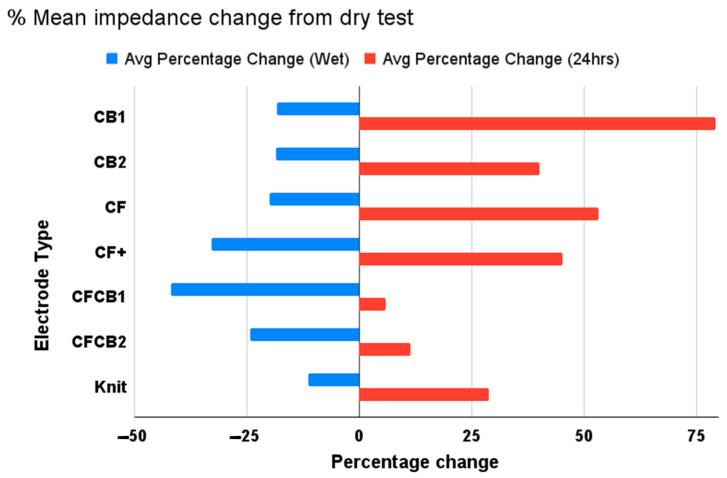
Percentage change in mean impedance between test conditions from baseline.

The mean impedance across different weights for different frequencies for the three different test conditions are visualized as heatmaps for all electrode types in Figure 14. The changes in impedance across different test conditions are listed as percentages (Figure 15). The characteristics of each dry electrode measured using the moisture management tester are detailed in Table 4, alongside the weight of electrodes before and after moisture exposure.

### 4.3. Electrocardiogram Signal-to-Noise Test

**Figure 16:**
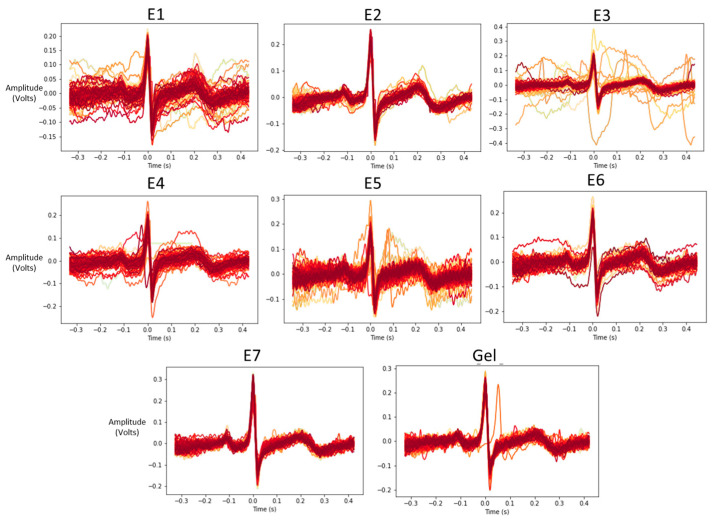
Beat-segmented ECG rhythms associated with each electrode type.

Individual ECG rhythm segments are synchronized around R-peaks to visualize the performance of each electrode type in Figure 16.

The SNR results for the different electrode types can be seen in Table 5.

## 5. Discussion

Reviewing the impedance characteristics, the mean impedance and frequency specific impedance had variations between electrodes under different test conditions. Under the dry test condition, as seen in Figure 11, knit electrode E7 demonstrated much lower impedance in all frequency bands, lower than even gel electrodes, with average impedance across frequency bands below 120 ohm. In embroidered electrodes, E5 (closely followed by E2 and E1) had low impedance characteristics across all frequencies. They were followed by comfort fill electrodes E3 and E4, which showed higher impedance characteristics. E6 showed the highest impedance among the electrodes, particularly in the frequency range of 20 Hz to 200 Hz.

After exposure to synthetic perspiration, there was a general decrease in impedance in all the dry electrodes, as observed in Figure 12 and Figure 15. The percentage reduction in impedance was higher for certain electrodes such as E6 (reduction of 45%), E4 (reduction of 30%), and E5 (reduction of 25%), indicating that certain embroidered structures were better at retaining moisture. The electrodes incorporating cross ball structures (E5, E2) had significantly lower impedance (below 100 ohm) after perspiration exposure compared to knit electrode E7 and comfort fill electrodes (E3 and E4), particularly in lower weight levels. The improved performance of the cross ball compared to the comfort fill electrode layout can be attributed to shorter conduction paths created by the radial stiches that efficiently connect the outer concentric layers to the inner layers. The interlocking of thread creates pockets and gaps to retain moisture. In contrast, the comfort fill electrode E3’s conduction path (other than occasional contact with adjacent horizontal rows) is typically longer, though perspiration can improve it. Comfort fill plus electrode E4, on the other hand, creates more gaps to retain moisture after exposure through uniform stitches along both axes.

Upon conducting the impedance test 24 h after drying the electrodes in the conditioning chamber, an increase in impedance characteristics for each electrode type is observed for almost all electrodes, as seen in Figure 13 and Figure 15. A slight increase was expected due to the lower relative humidity of 65% within the conditioning chamber and due to electrode oxidation from perspiration exposure. The increase in impedance, however, varies significantly across different dry electrode types in comparison to their baseline measurements the day before exposure to perspiration. Cross ball electrode E1 (increase of 75%) followed by comfort fill electrode E3 (increase of 50%), E4 (increase of 45%), and cross ball electrode E2 (increase of 40%) showed a considerably higher increase in impedance compared to the baseline measurement. While knit electrode E7 (increase of 25%) only shows a moderate increase in impedance, hybrid electrodes that combined comfort fill with cross ball 2 mm E5 (increase of 10%) and cross ball 1 mm E6 (increase of 5%) show only a mild increase from the baseline.

### 5.1. Contact Impedance and Load Weight

In all conditions, higher load weight reduces electrode impedance by stabilizing electrodes during measurements. However, it is important to note that increasing contact pressure can negatively affect user comfort when integrating such electrodes into sensing garments [8]. During the dry test (Materials and Methods, 3.4), the reduction in impedance with respect to load weight was lowest for knit electrode E7 owing to the multi-axial conducting property of yarn loops. The impedance of E7 is much lower than gel electrodes for load weights higher than weight level 4 (33.61 g), which is similar to findings made by Euler et al. [20].

Cross ball electrode designs (E1, E2, and E6) appear to benefit from load weight increase initially, though it provides a minor reduction after weight level 3 (23.61 g) compared to other dry electrodes. In contrast, the electrode combination of comfort fill and cross ball 1 mm (E5) shows the minimal change from its low impedance profile in weight level 1. This indicates that the electrodes with uneven surface contours perform better in lower weight levels compared to the uniform embroidered stitch pattern found in comfort fill electrodes (E3, E4). The contact force caused in response to load weight level appears to steadily decrease the impedance of comfort fill plus electrode E4.

The impedance measurements acquired immediately after perspiration exposure show a general reduction. However, the increase in weight after level 1 (3.61 g) had minimal influence on impedance measurements compared to the dry test. Influence of load weight appears to have been pronounced on comfort fill electrode E3 and cross ball electrode E1. Cross ball electrodes E2, E5, and E6 and knit electrode E7 show a minimal reduction after increasing weight compared to other electrodes. The steady reduction in impedance is observed in comfort fill plus electrode E4, similar to the dry test despite having much lower impedance at weight level 1. After 24 h of controlled drying within the conditioning chamber, a general increase in impedance in all dry electrodes is observed, though it still shows a similar reduction with load weight. Meanwhile, cross ball electrode E1 has a much higher impedance than comfort fill electrode E3, unlike the baseline measurement, particularly in frequencies below 1000 Hz in weight level 1. A steady impedance reduction is observed in comfort fill plus electrode E4 with load weights. Our findings validate some of the findings made by Kim et al. [40], where the impedance of different embroidered electrodes were compared on the basis of their skin–electrode impedance. The compared electrodes included a design similar to comfort fill (E3), concentric ring and wave-shaped electrodes. They report the wave-fill-shaped electrodes to have better performance, similar to the improved performance we observe with cross ball electrodes.

### 5.2. Moisture Management Test

The results from the moisture management test listed in Table 4 indicate key differences associated with the moisture transport capabilities of different dry electrodes. The wetting radius indicates how much of the surface area of the electrodes has been exposed. Top wetting time describes the duration needed to expose the top surface; meanwhile, bottom wetting time details the amount of time taken for moisture to wick through the electrode. While the knit electrode demonstrates the highest top wetting radius and minimal top wetting time, it shows no measured wetting at the bottom. This phenomenon can be attributed to the double-sided adhesive used to adhere it to the backing material. The lack of holes in the backing material in the knit electrode prevents moisture transportation through the electrode. The considerable reduction in impedance measured afterward indicates that the electrodes were exposed to the synthetic perspiration, nonetheless. The influences of holes on moisture transport can be observed in the different embroidered electrodes.

E6, followed by E1, shows a larger top wetting radius. E2 and E3 electrodes have a moderate top wetting radius, while E4 and E5 electrodes have the smallest top wetting radius. In terms of top wetting time, E4 took the largest time, followed by E6, E1, E3, and E2 electrodes. While all the embroidered dry electrodes had a similar bottom wetting radius, there is a difference between their bottom wetting times. E2, followed by E3, E4, E1, E5, and E6, have the highest bottom wetting times, indicated by the presence of more channels for moisture transport. Knit electrode E7, followed by E2, E5, E6, E4, E3, and E1, indicate a higher retention of moisture (electrode weight change) after 24 h of storage in a conditioning chamber. This indicates that gaps present between conductive yarns/threads trap and retain moisture which can eventually affect the electrode performance in capturing biopotential signals. The increase in dry electrode impedance after controlled conditioning can be attributed to the corrosive effect of perspiration similar to findings reported in prior studies [11,32]. The corrosive effect associated with the saline solution present within perspiration may result in the formation of an insulating layer over the silver thread due to residual perspiration solids (after liquids evaporate). These solids might have been retained due to the lack of mechanical agitation associated with washing machines resulting in the increase in dry electrode impedance.

### 5.3. ECG Signal Quality

The evaluation of the SNR ratio of different electrode types indicates that all dry electrodes developed can acquire electrocardiogram signals. However, the SNR measures based on QRS and TP sections indicate that electrode E7, followed by E3, E2, E4, E1, E6, and E5, have an SNR closest to gel-based electrodes. However, SNR measurements based on mean and standard deviation indicate E7, followed by E2, E6, E4, E5, E1, and E3, are closest to gel electrodes. The findings from the ECG SNR experiment indicate that ECG can be acquired from all the dry electrode types. However, knit-type electrodes show high SNR values, followed by E2 electrodes. The electrodes with lower SNR visually appear to have more variance in their beat-normalized ECG rhythms, as seen in Figure 13. Knit electrode E7, cross ball electrodes E2 and E6, followed by comfort fill electrodes E3 and E4, appear to have minimal variations in their normalized rhythms. Despite having higher impedance than other dry electrodes, E3 and E4 electrodes show a higher SNR. Similarly, despite having lower impedances in general, E6 and E5 electrodes show a lower SNR. This indicates that end signal quality measures alongside impedance should also be considered when evaluating such biopotential electrodes.

### 5.4. Limitations of the Study

It is important to acknowledge that electrodes were placed in the conditioning chamber after they were exposed to synthetic perspiration through the moisture management tester. Oxidative effects and mechanical agitations associated with washing machine cycles were not studied in this present work. The evaluation of the biopotential electrodes in this study was conducted with one participant for ECG acquisition. Integration of e-textile electrodes into different sensing garment can aid in understanding its effectiveness for other biopotential signals (EMG, EEG). In addition, future work can evaluate the change in resistance during the drying process or by obtaining additional impedance measurements at different relative humidity and temperature conditions. When using such dry e-textile electrodes for ambulatory physiological monitoring with different body types, it is important to understand the influence of contact pressures on signal quality. This study specifically evaluated the effect of perspiration exposure on such electrodes over a short-term (24 h) period. However, long-term stability of such dry electrodes was not investigated in this present study. We plan to inquire about the long-term stability of electrodes through a follow up study wherein the oxidative effects of moisture and perspiration along with the mechanical wear and tear associated with washing can be assessed using electron microscopy.

## 6. Conclusions

This paper involves the fabrication and electrical characterization of different dry e-textile electrodes using agar-based skin phantom before and after exposure to synthetic perspiration under different load weights. Through studying changes in impedance associated with different load weights, we can understand the tradeoff associated with different electrode types. We find considerable differences in their impedance characteristics due to perspiration exposure and moisture retention due to their structure. In general, we found that knit electrode E7, followed by E2 and E6 electrodes, perform well in terms of their impedance, moisture retention, and acquired ECG signal quality.

## Figures and Tables

**Table 1:** Summarized taxonomy and characteristics of dry biopotential electrodes.

Type	Fabrication Approach	Dimensions and Material	Surface Impedance (High to Low) [20–1000 Hz]	Biosignal Measured	Application Area	Mechanical Characteristics
Film	Inkjet Printing [16]	36 mm diameter (Polymer PEDOT: PSS)	500 kΩ to 250 kΩ	ECG	Wearable patch	Stretchable
	Laser Patterning [17]	24 mm diameter (Graphene)	13.8 kΩ to 445 Ω	ECG	Wearable patch	Stretchable, Permeable
Textile	Woven [18]	10 mm square (Silver polymer yarn)	200 kΩ to 3 kΩ	ECG	Clothing	Stretchable
	Embroidered [19]	20 mm diameter (Silver plated nylon)	400 kΩ to 800 Ω	EMG	Clothing	Flexible
	Knitting [20]	45 mm diameter (Silver polymer yarn)	5 kΩ to 400 Ω	Bio- impedance	Clothing	Stretchable
Sponge	Silicone- Molding [21]	10 mm diameter (Carbon fiber)	100 kΩ to 20 kΩ	EEG	Headcap	Water retention

**Table 2:** Thickness measurement associated with the dry e-textile electrodes.

Electrode Type	Minimum Thickness [µm]	Maximum Thickness [µm]	Mean Thickness [µm]
E1	1097.2	3225.8	1867.7
E2	1122.7	3235.96	1879.6
E3	1066.8	2880.36	1973.58
E4	1468.12	3266.4	2367.28
E5	1295.4	3855.72	1333.5
E6	1300.48	3296.92	1967.65
E7	1082.04	3154.68	2118.36

**Table 3:** Different weight levels and respective forces applied to each electrode.

Weight Level	1	2	3	4	5	6
Weight (g)	3.6	13.6	23.6	33.6	43.6	53.6
Force (N)	0.035	0.133	0.231	0.329	0.427	0.525

**Table 4:** Dry electrode characteristics associated with moisture management test.

Electrode	Electrode Weight Change [After Exposure] (g)	Top Wetting Radius (mm)	Top Wetting Time (s)	Bottom Wetting Radius (mm)	Bottom Wetting Time (s)
E1	0.092	15	3.781	5	11.969
E2	0.0204	10	0.188	5	14.657
E3	0.0099	10	2.156	5	13.437
E4	0.0114	5	6.86	5	12.781
E5	0.0195	5	3.282	5	11.938
E6	0.0134	20	6	5	8.543
E7	0.0296	25	0.187	0	61.437

**Table 5:** Signal-to-noise measurements associated with each electrode type.

Electrode Type	SNR QRS/TP (dB)	SNR Reg (dB)
E1	14.92672	12.04991
E2	15.48768	13.06902
E3	15.73079	11.38893
E4	15.31972	12.96527
E5	14.46775	12.70831
E6	14.91565	13.3378
E7	15.77633	15.41361
Gel	16.3097	16.72565

## Data Availability

Data can be made available to researchers upon request. The embroidered electrode design files for ZSK machine can be found in this cloud repository: https://drive.google.com/drive/folders/1Ne8cu-NY-8inm_aHNkLZrU8tWoPTCtGj?usp=sharing (accessed on 9 July 2023).
